# Perspective on strategies for matching across age and sex in physiology research: “recreationally active” is not good enough

**DOI:** 10.3389/fphys.2024.1517355

**Published:** 2025-01-07

**Authors:** Allyson M. Schweitzer, Daniel Fuller, Matthew D. Fliss, Cameron J. Mitchell

**Affiliations:** ^1^ School of Kinesiology, University of British Columbia, Vancouver, BC, Canada; ^2^ Department of Community Health and Epidemiology, University of Saskatchewan, Saskatoon, SK, Canada

**Keywords:** aging, exercise, matching, physical activity, self-report, sex comparisons

## Abstract

Cardiorespiratory fitness (CRF) and muscular fitness are powerful confounders in age and sex-related comparisons. This paper provides a perspective on the benefits and limitations of matching participants by physical activity behaviour, objectively measured fitness and normative fitness percentiles. Data presented herein are a subset of a larger study, and highlight that matching by physical activity, does not necessarily match on other metrics like physical fitness, especially when age-related comparisons are being made. Our data showed that young and older adults matched by physical activity behaviours showed the expected higher CRF and muscular fitness in male and younger participants, but older adults had higher CRF percentiles. This suggests that matching by physical activity behaviour may select older adults with relatively higher CRF. Researchers must choose their matching method carefully to ensure the appropriate aspects of fitness have been matched between groups. For clarity, they should also report when certain aspects of fitness have not been accounted for and give an explanation as to why.

## 1 Introduction

Confounding variables impact both dependent and independent variables in a study, potentially leading to false associations between these variables. Physical fitness is a common confounding variable in health-related outcomes and includes cardiorespiratory fitness (CRF), muscle strength, body composition and task performance. Physical activity, broadly defined as any movement requiring energy expenditure, is often used as a proxy for fitness. Both have significant effects on metabolic pathways, body systems (Fogelholm, 2010; Proper et al., 2011) and task performance, and therefore must be controlled for in research studies.

To illustrate physical fitness’ confounding role, consider a study recruiting young untrained females to assess two exercise training protocols. If training status is determined by asking participants if they engage in physical activity more than once a week, one group might be less fit due to this simplistic measure. Untrained individuals respond more to exercise training than trained individuals (Plowman et al., 1979), therefore, this could lead to a false conclusion that one exercise protocol is superior. To control for this, the baseline fitness of participants should be rigorously matched.

Matching aims to equate covariate distribution between groups and is commonly used in epidemiology (Stuart, 2010). Various forms of matching exist, such as one-to-one, paired, and propensity score matching (Stuart, 2010). The purpose is to ensure that participants in different groups are similar on average, concerning characteristics believed to be confounders in the association between the independent and dependent variables (Faresjö and Faresjö, 2010; Stuart, 2010). In the case of the two groups of females, physical fitness can be matched using questionnaires, or more accurate measures of fitness like VO_2peak_. VO_2peak_, the gold-standard measure of CRF, is measured via maximal graded exercise tests. It may also be estimated with a submaximal test, which is useful for populations that are unable to complete maximal exercise, but is more prone to error (Dugas et al., 2023).

Matching fitness is more challenging when comparing individuals of different ages and sexes, as both age and sex influence fitness. Muscular fitness and CRF decline with age (Goodpaster et al., 2006; Plowman et al., 1979), and males typically have greater CRF and muscular fitness than females (Bishop et al., 1987; Tarnopolsky, 1998). Furthermore, older adults have lower average physical activity levels than young adults (Hallal et al., 2012) and lower average fitness for a given level of physical activity (Plowman et al., 1979).

Individuals can be matched for physical activity and fitness across ages or between sexes by subjective or objective assessments. Objective matching is challenging due to age and sex effects on fitness, therefore subjective assessments are appealing. Untrained volunteers are commonly matched on subjective physical activity classifications such as self-reported weekly minutes of physical activity, or metabolic equivalent (MET) minutes. This method assumes that if physical activity is matched, so is physical fitness. For example, Hart et al., 2019, investigated age-related changes in the unfolded protein response to a single bout of resistance training, matching participants on the criterion of “not participating in routine exercise training for more than 2 days per week.” Similarly, trained volunteers are recruited based on self-reports such as participating in structured training for a minimum time (Petré et al., 2021). Self-reports are prone to error (Prince et al., 2008) and matching becomes more difficult when groups differ by age *and* sex.

Current matching methods have many limitations, especially when matching across age *and* sex. Matching individuals based on age and sex-stratified fitness percentiles (from objective fitness tests) may offer a more valid way of matching physical fitness between groups. This method involves volunteers completing objective fitness tests, and classification into age and sex-stratified percentiles. This method addresses age and sex’s confounding effects on physical fitness, removes self-report error and may offer an improved method of comparing individuals across age *and* sex.

The data presented herein, are a subset of a larger study that aimed to investigate age-related differences in how skeletal muscle responds to an acute bout of resistance exercise. In this larger study, both physical activity and physical fitness were matched between young and older adults. In doing so, it was observed that matching on one fitness variable (i.e., physical activity) does not necessarily match on the others (i.e., physical fitness). This perspective presents the data subset from our larger study as evidence for the importance of intentional matching when making age and sex-related comparisons in health research.

## 2 Materials and methods

### 2.1 Participants

This study was approved by the University of British Columbia Research Ethics Board (H22-01203). Written informed consent was received from all participants. Healthy young (19–30 years) and older (65–85 years) male and female volunteers were recruited from the Vancouver, British Columbia, area. Participants were recreationally active, and self-reported participating in no more than 2 h per week of structured, moderate-vigorous lower-body resistance or aerobic training in the last 6 months.

### 2.2 Experimental procedures

Participants completed a maximal CRF ramp test on a cycle ergometer (Monark, LC6, Sweden) to measure VO_2peak_ (mL/min/kg). They cycled to volitional fatigue and VO_2peak_ was recorded as the highest 30-second average VO_2_. Maximal grip strength was tested by handgrip dynamometer (Handeful, Digital Hand Dynamometer) following methods in Hoffmann et al. (2019). Grip strength (kg) was recorded as the sum of the right and left hands. Primary outcomes were VO_2peak_ (mL/min/kg), grip strength (kg) and VO_2peak_/grip strength age-sex stratified percentiles. Volunteers completed the International Physical Activity Questionnaire (IPAQ). Young adults completed the long form and older adults the elderly short form. MET-minutes per week were calculated from the IPAQ but did not determine eligibility.

### 2.3 Matching variables: fitness percentile line of best fit extrapolation

To create a matching variable, age and sex-stratified fitness percentile lines of best fit were created using the findings from Kaminsky et al., 2022, for VO_2peak_ and Hoffmann et al., 2019, for grip strength. Participants’ VO_2peak_ (mL/min/kg) and grip strength (kg) scores were used to extrapolate their age and sex-stratified fitness percentile using these lines of best fit. If participants’ extrapolated percentile fell below 1, they were assigned the 1^st^ percentile. If their percentile fell above 100, they were assigned the 99^th^ percentile.

### 2.4 Statistical analysis

All statistical analyses were conducted using GraphPad Prism version 10.2.3 for Mac OS X (GraphPad Software, Boston, MA). Two-way ANOVA (age × sex), followed by Fisher’s LSD test was performed on all metrics. Data are presented as mean ± standard deviation.

## 3 Results

### 3.1 Participants

13 young females (22.5 ± 2.9 years), 16 young males (21.5 ± 2.3 years), 10 older females (74.4 ± 3.7 years) and 9 older males (71.2 ± 2.4 years) were recruited. Males weighed more (*p* = <0.0001) and were taller than females (*p* = <0.0001). Older females (22.3 ± 2.1 kg/m^2^) had significantly lower BMI than older males (24.67 ± 1.94 kg/m^2^, *p* = 0.025). No other significant differences in BMI were demonstrated. No significant differences in IPAQ scores were demonstrated between groups.

### 3.2 Matching on self-report fitness, age, and sex

**FIGURE 1:**
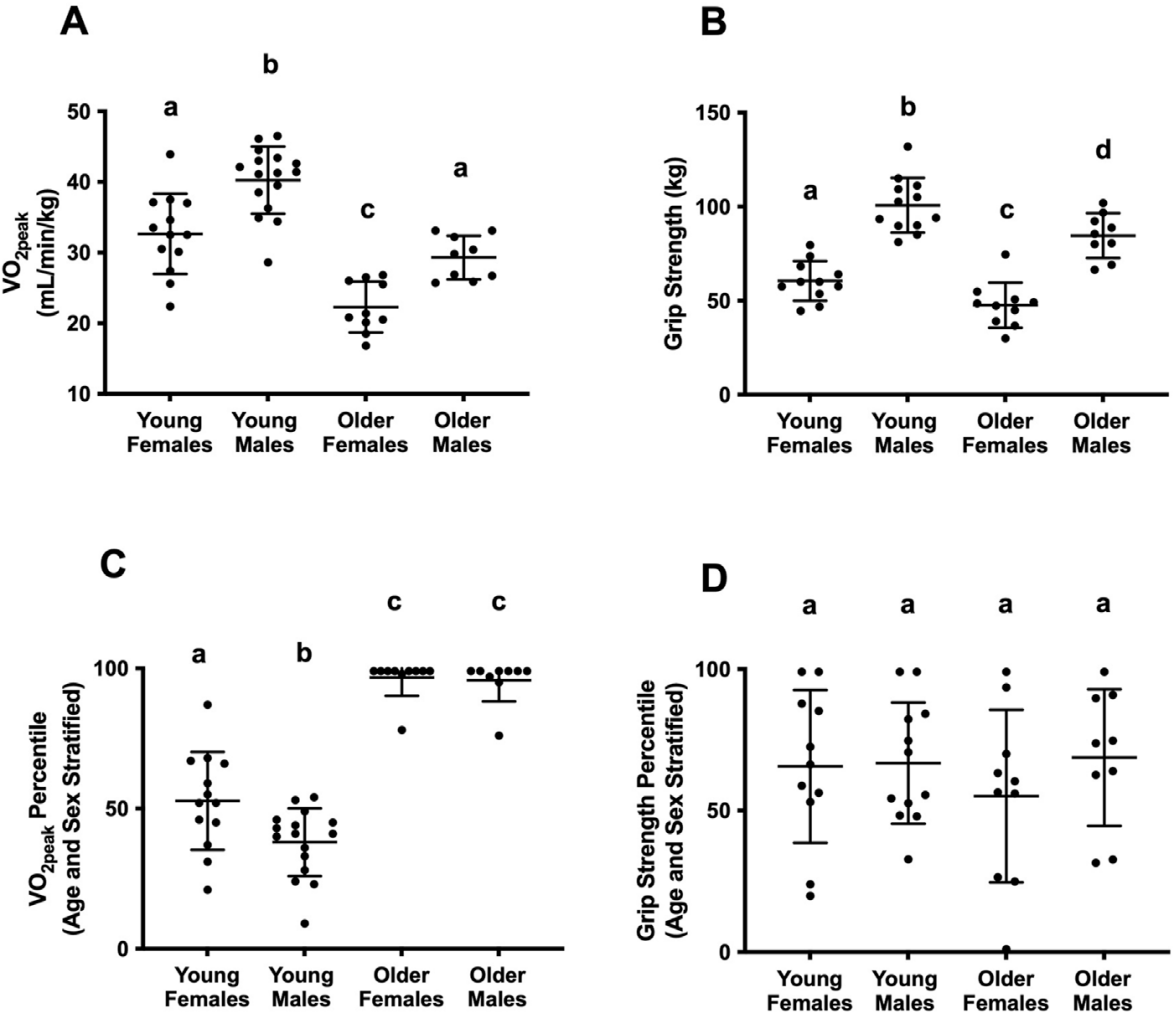
**(A)** Measured VO_2peak_ (mL/min/kg) between ages and sexes. **(B)** Measured grip strength (kg) between ages and sexes. **(C)** Comparison of age and sex-stratified cardiorespiratory fitness (VO_2peak_) percentiles. **(D)** Comparison of age and sex-stratified muscular fitness (grip strength) percentiles. Different letters above each group’s data denote statistically different post-hoc differences between groups (*p* < 0.05). Groups with the same letter are not statistically different from one another.

Groups matched for self-reported physical activity, age, and sex, showed expected results based on the existing literature (Bishop et al., 1987; Diaz-Canestro et al., 2022). Young adults had greater VO_2peak (_36.86 ± 6.38 mL/min/kg, *p* = <0.001) (Figure 1A) and grip strength (81.51 ± 24.05 kg, *p* = <0.001) (Figure 1B) than older adults (25.62 ± 4.87 mL/min/kg, 65.11 ± 22.28 kg). Males had greater VO_2peak_ (36.32 ± 6.79 mL/min/kg, *p* = <0.001) (Figure 1A) and grip strength (93.81 ± 15.53 kg, *p* = <0.001) (Figure 1B) than females (28.15 ± 7.11 mL/min/kg, 54.37 ± 12.86 kg).

### 3.3 Matching on relative fitness, age, and sex

Matching relative fitness score, age, and sex showed important differences compared to matching based on self-reported data (≤ 2 hours structured lower-body exercise per week). Older adults had higher VO_2peak_ percentiles (96.32 ± 6.7) than young adults (44.66 ± 15.98, *p* = <0.0001) and young females (52.77 ± 16.79) had higher VO_2peak_ percentiles than young males (38.06 ± 11.7, *p* = 0.003) (Figure 1C). No differences in VO_2peak_ percentiles were observed between older males (95.78 ± 7.11) and females (96.8 ± 6.27, *p* = 0.86). No differences in grip strength percentiles were observed between age groups (*p* = 0.6) or between sexes (*p* = 0.36) (Figure 1D).

## 4 Discussion

As expected, recruiting individuals who engage in less than 2 hours of lower-body exercise weekly showed higher muscular (grip strength, kg) and CRF (VO_2peak_, mL/kg/min) in younger relative to older adults and males relative to females. When using normative data to compare fitness percentiles, however, older adults showed higher CRF but similar muscular strength, highlighting the limitations of matching CRF across age groups by self-report. Matching participants on stratified fitness percentiles can effectively control for performance and behaviour differences influenced by age. Understanding the strengths and weaknesses of each matching method (Table 1) will facilitate informed decision-making in research design.

**TABLE 1:** Advantages and disadvantages of each matching method. The focus is on comparison groups for which each method is valid. Cardiorespiratory fitness (CRF).

Method of matching physical fitness	Advantages	Disadvantages
Measured Fitness (Performance Capacity)	• Matching individuals of the same age and sex (CRF and muscular)	• Matching individuals of different ages or sexes (CRF and muscular fitness)
Physical Activity Minutes (Behaviour)	• Matching older adults (CRF/grip strength) • Matching young adults (grip strength) • Ease of recruitment	• Matching individuals of different age groups (CRF) • Matching between sexes in young adults (CRF) • Assumes physical activity is a linear correlate of physical fitness
FFM-Normalized Fitness (Tissue/Muscle-Specific)	• Matching young male and female skeletal muscle (Tripp et al., 2024)	• Matching whole-individuals • Possibly matching between young and older adults (Fleg et al., 2005)
Fitness Percentiles (Whole-Individuals)	• Matching individuals of different ages and sexes (CRF and grip strength)	• Relies on the availability of normative data • Ease of recruitment

Objectively measuring fitness is valid for same age and sex comparisons. This method is impractical, however, when groups differ by age and/or sex. For example, a 30-year-old male with a VO_2peak_ of 22.6 mL/min/kg falls within the 20^th^ percentile for his age group, whereas a 70-year-old male with the same VO_2peak_ falls between the 80^th^ percentile, and a 70-year-old female falls above the 99^th^ percentile (Kaminsky et al., 2022). In this case, a very unfit young male would be recruited, and a very fit older male/female would be recruited, leading to possible confounding effects of physical fitness. Similar issues arise when matching muscular fitness scores between individuals of different ages and/or sexes (Hoffmann et al., 2019). It is also important to maintain consistency in the method used to measure objective fitness scores with the methodology of the reference data. VO_2peak_ must be measured using a maximal test, however, the results from submaximal tests are commonly extrapolated and reported as VO_2peak_ (Hoffmann et al., 2019). Due to systemic bias in CRF estimates from submaximal protocols (Dugas et al., 2023), it is not appropriate to match objectively measured and estimated CRF data within a single study. Further, the exercise mode of the test used will influence CRF values. For example, an individual will typically achieve a higher VO_2peak_ score on a maximal treadmill test than a maximal cycle ergometer test (McArdle et al., 1973). Thus, where possible, the CRF testing methodology should match the methods of the reference data as closely as possible.

Self-reported physical activity is advantageous for its simplicity–no physiological tests are required, facilitating recruitment. This method assumes that matching physical activity is appropriate for age and sex comparisons as individuals do not need to have the same measured fitness. Our findings challenge these assumptions. Young and older males and females who report similar weekly physical activity minutes do not fall within the same CRF percentiles (determined by VO_2peak_ test) (Figure 1C). Young adults have significantly lower CRF percentiles than older adults and young females have significantly higher CRF percentiles than young males (Figure 1C). Self-report data is prone to error (Prince et al., 2008), therefore, older adults may underestimate and/or young adults (and to a greater extent, young males) may overestimate their physical activity levels. These findings suggest that matching physical activity minutes effectively matches CRF between sexes in older adults, but not between sexes in young adults or different age groups. These findings are specific to CRF, as individuals of different ages and sexes matched on physical activity minutes, show no differences in grip strength percentiles (Figure 1D), suggesting this matching method is valid when grip strength is a potential confounder.

A final method to be discussed is matching FFM-normalized fitness between groups. Variations in body fat percentage between males and females influence physical fitness (Tarnopolsky, 1998), thus matching FFM-normalized fitness aims to eliminate this confounding variable. This method is effective when comparing young male and female muscle (Tripp et al., 2024), however, Fleg et al., 2005, demonstrated that VO_2peak_/FFM declines more rapidly in older adults (like VO_2peak_/kg bodyweight). Thus, using this method to match between age groups might cause the same findings as matching performance-based outcomes (fitness percentiles differing significantly between groups, where older adults score higher than young adults). Matching FFM-normalized fitness assumes that body composition is the only confounder of physical fitness. However, other systemic differences contribute to overall fitness as well. Therefore, this method effectively matches physical fitness when outcomes are tissue-specific but is not necessarily valid for other outcome measures. The concern of matching *whole individuals* can be addressed by matching fitness percentiles. Matching fitness percentiles addresses whole-body fitness, rather than body composition differences only.

Each of the matching methods discussed has advantages and disadvantages, and their suitability depends on the comparison group, research question and research outcomes (Table 1). The challenges of matching physical fitness across multiple variables are clear and no universal strategy is optimal in all situations. Instead, it emphasizes that different aspects of physical fitness are controlled depending on the method chosen, and not all methods are valid for all population comparisons (Table 1).

Matching fitness percentiles should be considered when making age and sex comparisons where physical fitness is a confounding variable. This method, however, has limitations, including the need for normative datasets that are not always available and may be context-specific. As mentioned, different methods of collecting the same objective fitness scores have different measurement errors and biases. As objective fitness scores are needed to create fitness percentiles, it is important to match, as closely as possible, the methods used by the normative dataset to the methods used in the present study. These challenges are even greater when assessing muscular fitness as, beyond standardized measurements of grip strength, there is little consistency in measurement protocols for strength across different demographics and research areas. The use of normative data can also introduce error if its population is biased or unrepresentative. For example, the present study used normative data available from the United States, rather than Canada because Canadian normative data used a submaximal test to measure CRF (Hoffman et al., 2019), and the present study used a maximal exercise test. Given the similarities between Canadian and United States demographics, and due to the error of submaximal tests, it was decided that it was more important to match the CRF measurement method than to match the population. Researchers should be aware of the extent to which geographical characteristics affect their outcomes when choosing normative datasets. The small sample size of the present study limits the precession of the findings but still illustrates the trade-offs associated with choosing each matching method. As the current study was part of a larger study investigating age-related differences in skeletal muscle responses to exercise in Vancouver, Canada, applying these matching methods to a larger geographically diverse sample could offer better insights into age and sex-based matching.

Various methods for matching physical fitness between groups have been described, each addressing different aspects of fitness. Performance capacity can be matched with objective fitness tests (i.e., measured VO_2peak_), behavioural aspects with self-reports (i.e., number of physical activity minutes per week) and muscle/tissue composition differences with FFM-normalized fitness tests. Matching fitness percentiles offers the advantage of matching on average, individuals’ whole-body fitness and should be considered when age and sex comparisons are made. Each method has merits and drawbacks, researchers must understand both when choosing their matching criteria. Researchers should also explicitly acknowledge which aspects of fitness have/have not been addressed by their chosen method and justify it in their reports. Careful selection of matching criteria and transparency in reporting will enhance the validity and reliability of research outcomes.

## Data Availability

The raw data supporting the conclusions of this article will be made available by the authors, without undue reservation.
